# Effect of Preoperative Opiate Use on Outcomes After Posterior Lumbar Surgery

**DOI:** 10.7759/cureus.22663

**Published:** 2022-02-27

**Authors:** Alex Mierke, Omar Ramos, Jun Chung, Wayne K Cheng, Olumide Danisa

**Affiliations:** 1 Orthopaedic Surgery, Loma Linda University Medical Center, Loma Linda, USA; 2 Orthopaedics, Loma Linda University Medical Center, Loma Linda, USA; 3 Orthopaedics, Jerry L. Pettis Veterans Affairs Medical Center, Loma Linda, USA

**Keywords:** spine, readmission, smoking, lumbar-fusion, opioids use

## Abstract

Introduction

The prescription opioid epidemic and widespread use of narcotic medications have introduced new challenges when treating patients undergoing spine surgery. Given the ubiquity of preoperative opioid consumption amongst patients undergoing spine surgery, further research is needed to characterize perioperative risks. Our goal is to compare outcomes following primary lumbar decompression, instrumentation, and fusion based on preoperative opioid prescriptions.

Methods

Patients older than 18 years of age who underwent a primary one- to two-level lumbar decompression, instrumentation, and fusion were included in the study. Patients with known malignancy, surgery involving three or more lumbar levels, current or previous use of neuromodulation, revision surgery, anterior or far lateral interbody fusions, acute fractures, or other concurrent procedures were excluded. Patients were divided into chronic opioid therapy (COT; over six months), acute opioid therapy (AOT; up to six months), and opiate-naïve groups. Opioid prescriptions, demographics, smoking status, readmission rates within one year, and reoperation rates within two years were recorded based on electronic medical record documentation.

Results

Out of 416 patients identified, 114 patients met the inclusion criteria. Thirty-eight patients (33.3%) were on COT, 38 patients (33.3%) were on AOT, and 38 patients (33.3%) were opioid naïve preoperatively. Readmission rates within one year for COT, AOT, and opioid naïve patients were 34.2%, 26.3%, and 10.5%, respectively (p=0.047). Reoperation rates within two years for COT, AOT, and opioid naïve patients were 34.2%, 15.8%, and 13.2%, respectively (p=0.049). We also found current or former smokers were more likely to be on AOT or COT than never smokers (78.4% vs. 57.1%; p=0.017).

Conclusion

Long-term opiate use is associated with an increased risk for readmission within one year and revision within two years. Physicians should discuss the increased risks of readmission and revision surgery associated with lumbar decompression and fusion seen in patients on preoperative opioid therapy.

## Introduction

In the United States, the widespread use of opioid medications has created significant challenges for clinicians and patients. According to the 2019 National Survey on Drug Use and Health, two million people were diagnosed with an opioid use disorder in 2018 [1]. In 2018, there were 67,367 drug overdose deaths in the United States, two-thirds (46,802) of those were due to opioids [2].

Over the past five years, multiple database studies have highlighted important associations between preoperative opioid use and surgical outcomes. Preoperative opioid use is associated with increased length of stay, increased 90-day readmission rates, implant-related complications, infection rates, pulmonary insufficiency, and increased healthcare-related costs [3-5]. These studies point to the dangers of chronic opioid therapy and its impact on clinical outcomes.

Nearly all previous studies rely on large databases to collect clinical and patient-reported outcomes. While this provides well-powered studies, limitations of a database study include reliance on administrative claims, coding methodology, and the inability to exclude patients who were prescribed opioids for unrelated problems [4,6]. The purpose of this study is to continue to characterize the impact that chronic opioid use has on surgical outcomes following one- or two-level lumbar decompression and fusion.

## Materials and methods

After the Human Investigation Committee ( Institutional Review Board - IRB) of Loma Linda University approved this study (IRB#5190139), the authors performed a retrospective review of the medical records of all patients who underwent lumbar decompression and fusion at a tertiary referral center from 2013-2017. Inclusion criteria included patients older than 18 years of age who underwent a primary one- or two-level posterior decompression (laminectomy or laminotomy) with either posterolateral intertransverse arthrodesis (PLF), transforaminal lumbar interbody fusion (TLIF), or posterior lumbar interbody fusion (PLIF). Exclusion criteria included patients without at least two years of follow up, patients undergoing revision surgery, patients with known malignancy (neoplasm of the spine or other body sites), surgery involving three or more levels, patients with current or previous use of neuromodulation (dorsal root ganglion stimulator or spinal cord stimulator), patients who required iliac crest bone graft harvest during surgery, anterior (ALIF) or far lateral interbody fusions (DLIF), patients with acute fractures or patients undergoing other concurrent procedures.

The definition of chronic opioid therapy (COT) varies significantly in the literature [7,8]. A 2019 study by Oleisky et al. [9] reviewed six definitions of preoperative opioid use in the literature and found that continuous use over six months was most predictive of postoperative satisfaction and patient-reported outcomes. Therefore, we utilized this definition for COT in our study. Patients were divided into chronic opioid therapy (COT; over six months), acute opioid therapy (AOT; up to six months), and opioid-naïve groups based on preoperative opioid usage. Opioid type, dose, frequency, and route were converted to milligram morphine equivalent (MME) dose. Primary outcomes included visual analog scale (VAS) scores, nicotine use, readmission rates within one year, and reoperation rates within two years.

VAS is a common, single-unit outcome measure for leg and back pain. The original description involves a 10 cm line with the numbers 0-10 associated with increasing pain scores from "no pain" to "unbearable pain". VAS scores were recorded for back and leg pain prior to surgery as well as between 6-12 months postoperatively. Data is presented as a "delta" VAS (final - initial) with negative numbers associated with improvement in pain. Nicotine use was recorded as "current", "former", or "never" users based on smoking history documented in the electronic medical record (EMR). Any readmission or subsequent surgery was recorded along with the underlying reason as documented in the EMR.

Categorical data was compared using chi-squared tests, and continuous variables were compared using analysis of variance (ANOVA) tests. Statistical analysis was conducted using SPSS 21.0 (IBM Inc., Armonk, USA).

## Results

**Figure 1:**
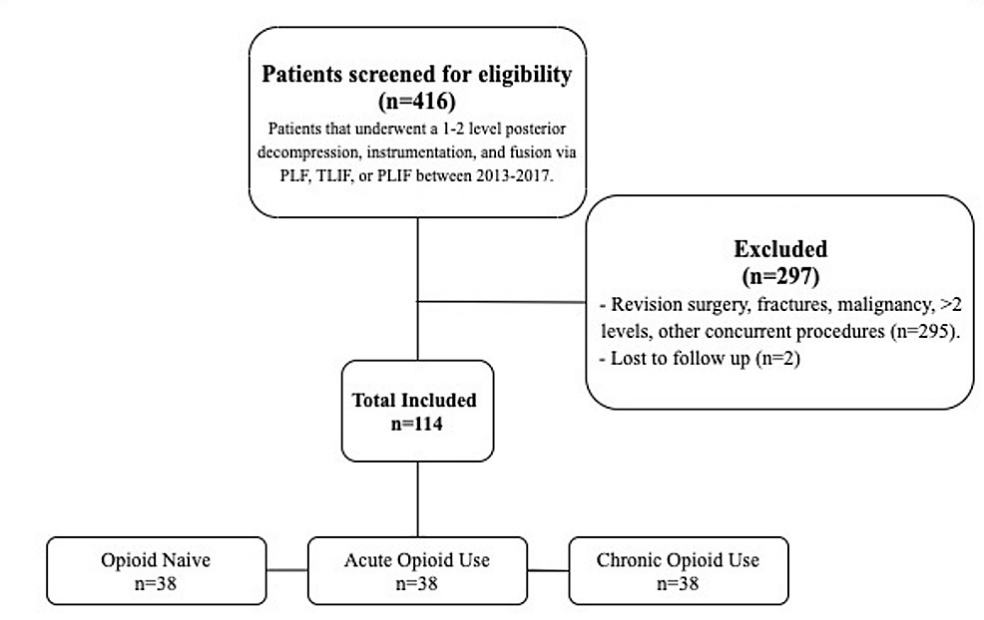
Patient flow diagram PLF - posterolateral intertransverse arthrodesis; TLIF - transforaminal lumbar interbody fusion; PLIF - posterior lumbar interbody fusion

Initial review of patient data yielded 416 patients who met inclusion criteria by undergoing a one to two-level posterior decompression, instrumentation, and fusion via PLF, TLIF, or PLIF between 2013 and 2017. Of the 416 patients, 297 were excluded because they were associated with revision surgery, fractures, malignancy, three or more levels, anterior or far lateral interbody fusions, and/or other concurrent procedures. Two patients in each group were lost to follow-up (5.3%; Figure 1). 

Table 1 shows the demographic characteristics of the 114 patients included in the final analysis.

**Table 1:** Demographic characteristics

	Opioid naïve (n=38)	Acute opioid use (n=38)	Chronic opioid use (n=38)	p-value
Age at surgery: mean (SD)	62.7 (12.8)	58.7 (17.4)	65.2 (10.6)	0.119
Gender: n (%)				0.373
Female	27 (71.1%)	22 (57.9%)	27 (71.1%)	
Male	11 (28.9%)	16 (42.1%)	11 (28.9%)	
BMI: mean (SD)	30.1 (6.0)	29.7 (5.4)	29.9 (6.8)	0.952
Levels: n (%)				0.881
One level	25 (65.8%)	21 (55.3%)	23 (60.5%)	
Two levels	13 (34.2%)	17 (44.7%)	15 (39.5%)	
Smoking status: n (%)				0.028
Never smoker	27 (71.1%)	20 (52.6%)	16 (42.1%)	
Former smoker	7 (18.4%)	11 (28.9%)	19 (50%)	
Current smoker	4 (10.5%)	7 (18.4%)	3 (7.9%)	
Ethnicity: n (%)				0.861
White	25 (65.8%)	25 (65.8%)	28 (73.7%)	
Hispanic	9 (23.7%)	10 (26.3%)	5 (13.2%)	
Asian	2 (5.3%)	1 (2.6%)	2 (5.3%)	
African American	2 (5.3%)	2 (5.3%)	2 (5.3%)	
Middle Eastern	0 (0%)	0 (0%)	1 (2.6%)	
Visual analog scale (VAS)				
Back pain	-1.68 (2.21)	-2.18 (2.53)	-1.29 (1.16)	0.171
Leg pain	-5.39 (1.70)	-4.61 (2.14)	-4.18 (1.61)	0.016

Current or former nicotine users were more likely than non-users to be on acute or chronic opioid therapy (78.4% vs. 57.1%, p=0.017; see Table 2).

**Table 2:** Smoking status and opioid use

Smoking status: n (%)	Opioid naïve	Acute or chronic opioid use	p-value
Never smoker	27 (42.9%)	36 (57.1%)	0.017
Current or former smoker	11 (21.6%)	40 (78.4)	

The delta VAS scores for leg pain for COT, AOT, and opioid naïve patients were -4.18, -4.61, and -5.39, respectively (p=0.016). The delta VAS scores for back pain for COT, AOT, and opioid naïve patients were -1.29, -2.18, and -1.68, respectively (p=0.171). Patients who were opioid naïve received significantly more leg pain relief following surgery, but there was no significant absolute difference in back pain.

Given the known association between nicotine use and nociception, VAS scores were compared to nicotine use (Table 3). The delta VAS scores for back pain for never smokers, previous smokers, and current smokers were -1.94, -1.73, and -0.71, respectively (p=0.004). Similarly, the delta VAS scores for leg pain for never smokers, previous smokers, and current smokers were -4.97, -4.68, and -3.79, respectively (p=0.044). Due to this association, stratified analysis was performed to eliminate confounding variables.

**Table 3:** Visual Analogue Scale and nicotine use VAS - Visual Analog Scale

Delta VAS: mean (SD)	Never smoker (n=63)	Former smoking (n=37)	Current smoker (n=14)	p-value
Back pain	-1.94 (2.12)	-1.73 (1.73)	-0.71 (0.99)	0.004
Leg pain	-4.97 (1.80)	-4.68 (2.31)	-3.79 (2.19)	0.044

Amongst never-smokers, the delta VAS scores for leg pain for COT, AOT, and opioid naïve patients were -4.56, -4.90, and -5.26, respectively (p=0.53). Amongst former smokers, delta VAS scores for leg pain for COT, AOT, and opioid naïve patients were -3.95, -4.73, and -6.57, respectively (p=0.032). Amongst current smokers, delta VAS scores for leg pain for COT, AOT, and opioid naïve patients were -3.67, -3.57, and -4.25, respectively (p=0.897). Only former smokers had a statistically significant decrease in delta VAS scores for leg pain.

The readmission rate between opioid non-users, acute users, and chronic users was 10.5%, 26.3%, and 34.2%, respectively (p=0.047). Table 4 shows opioid usage versus readmission and reoperation rates. 

**Table 4:** Opioid usage and readmission/reoperation rate

Readmission/reoperation rate: n (%)	Opioid naïve	Acute opioid use	Chronic opioid use	p-value
Readmission	4 (10.5%)	10 (26.3%)	13 (34.2%)	0.047
No readmission	34 (89.5%)	28 (73.7%)	25 (65.8%)	
Reoperation	5 (13.2%)	6 (15.8%)	13 (34.2%)	0.049
No reoperation	33 (86.8%)	32 (84.2%)	25 (65.8%)	

The reasons for readmission directly related to spine surgery included infection (n=7, 25.9%) and/or need for further surgery (n=14, 51.2%). Other reasons for admission that were indirectly related to spine surgery included stroke (n=1, 3.7%) gastrointestinal bleed (n=1, 3.7%), symptomatic anemia (n=1, 3.7%), deep vein thrombosis (DVT; n=1, 3.7%), perianal abscess (n=1, 3.7%) and pneumonia (n=1, 3.7%).

Causes for reoperation included infection (n=3, 12.5%), pseudarthrosis (n=6, 25%) adjacent segment disease (n=3, 12.5%) recurrent stenosis at another level (n=7, 29.2%) and/or implant migration (n=5, 20.8%).

## Discussion

In the current study, the rate of readmission of opioid naïve, acute users, and chronic users was 10.5%, 26.3%, and 34.2%, respectively (p=0.047). The rate of reoperation between opioid naïve, acute users, and chronic users was 13.2%, 15.8%, and 34.2%, respectively (p=0.049). These findings are similar to those in recent studies, which also found a correlation between readmission or reoperation rate and history of opioid use [4,5,10-13].

Increased readmission and reoperation rates ultimately lead to increased healthcare costs. In a prospective longitudinal registry study, Sivaganesan et al. [14] found that, among other variables, pre- and postoperative opioid use, readmission rates, and postoperative health care visits play an important role in the overall cost for elective spinal surgery. Jones et al. [15] found that the mean hospital revenue per day for geriatric patients with an opioid-related adverse drug event was $3,076 less than patients without such an event. As it becomes more important to practice cost-conscious medicine, physicians should be aware of the increased risk of complications in opioid-dependent patients. Patients on chronic opioid therapy can be expected to place a larger burden on health care resources than their opioid naïve counterparts. Physicians are encouraged to maximize conservative management prior to surgery and to counsel patients preoperatively on their increased risk of readmission and reoperation.

Preoperative opioid use was associated with less improvement in postoperative leg pain but no difference in postoperative back pain relief (see Table 1). To eliminate nicotine use as a confounding variable, a stratified analysis was performed (Table 5). This analysis continued to demonstrate the negative association between preoperative opioid use and postoperative leg pain relief. This data is consistent with recent studies which demonstrate increased postoperative opioid requirements for patients using opioids preoperatively [16,17]. Given that patients on chronic preoperative opioid therapy perceive less pain improvement following surgery, clinicians operating on this subset of patients can expect worse outcome measures, regardless of technical ability.

**Table 5:** Visual Analog Scale (for leg pain) and opioid use stratified by nicotine use VAS - Visual Analog Scale

Delta VAS for leg pain: mean (SD)	Opioid naïve (n=38)	Acute opioid use (n=38)	Chronic opioid use (n=38)	p-value
Never smoker (n=63)	-5.26 (2.09)	-4.9 (2.07)	-4.56 (1.65)	0.529
Former smoker (n=37)	-6.57 (2.37)	-4.73 (2.49)	-3.95 (1.84)	0.032
Current smoker (n=14)	-4.25 (2.5)	-3.57 (2.64)	-3.67 (0.58)	0.897

Current or former nicotine users were more likely than non-users to be on acute or chronic opioid therapy. This is supported by recent studies, which also found a correlation between nicotine and opioid dependence [18,19]. Previous studies have suggested that prolonged nicotine exposure is associated with structural damage to organ systems (osteoporosis, spinal degenerative disc disease, impaired bone, and wound healing), altered pain processing, personality disorders, and substance abuse [18,20]. Patients with a history of nicotine dependence are more likely to be on chronic opioid therapy and have diminished bone quality. Poor bone quality is associated with decreased screw pullout strength [21], pseudarthrosis [22], increased risk of hardware failure [23], adjacent level disc degeneration [24], and proximal junctional kyphosis [25], all of which can lead to reoperation or readmission. Physicians should be aware of these correlations and their impact on postoperative pain management requirements, readmission rates, and reoperation rates.

As the literature continues to demonstrate associations between preoperative opioid use and worsened outcomes, efforts to limit or wean preoperative opioids have been proposed. However, given the psychosocial complexity of chronic pain and opioid dependence, clinical studies are complicated by difficulties with randomization, noncompliance, and patient dropout. Current guidelines for opioid weaning, as described by Manchikanti et al., involve a 10% reduction in opioids per week [26]. Furthermore, evidence from Lally et al. demonstrates that changing behavioral habits requires 12 weeks, with significant variation noted between individuals [27]. These studies indicate that opioid weaning trials can take a minimum of three months to implement, with high levels of relapse and noncompliance. Jain et al. reported fewer adverse events in patients that ceased opioid medications three months prior to surgery [4]. Nguyen et al. matched 41 patients on chronic opioid therapy (more than four weeks) with 41 opioid naïve patients and found that patients who weaned from opioids had similar patient-reported outcomes to opioid naïve patients [28]. More research is needed to establish a safe and effective preoperative opioid weaning program and the impact that such a plan would have on surgical and patient-reported outcome measures [29].

One limitation of this study is that it is a retrospective chart review that introduces possible errors in data input from practitioners and inaccuracy in patient-reported opioid dosages and durations. Furthermore, opioid dosage and duration were based on practitioner prescriptions but may not accurately reflect the actual prescriptions filled or medications taken. Also, we were not able to evaluate disease severity before surgery, which could affect the need for opioids before and after surgery. Recent database studies have taken advantage of insurance claims to monitor actual prescriptions filled, which we were unable to perform in our study. However, our study was able to look at more granular detail that International Classification of Diseases (ICD) 9 and 10 coding definitions lack, such as individual levels of surgery, approach, radiographic parameters, changes in patient-reported outcome measures such as VAS scores, and surgical/anesthesia notes. Loss to follow-up bias could have potentially affected the internal validity of our results. However, we found a loss of follow-up of only two patients in each group after applying the inclusion and exclusion criteria. This led to a loss of follow-up of 5.3% in each group. Previous studies have demonstrated that <5% loss leads to little bias, while >20% poses a serious threat to validity [30].

## Conclusions

Chronic opioid use is associated with worse patient-reported outcome measures, increased risk for readmission within one year, and increased revision rate within two years following a primary posterior lumbar decompression, instrumentation, and fusion. Smokers are more likely to be chronic opioid users. Given the prevalence of opioid usage in the U.S. population, physicians should continue to educate acute and chronic opioid users about their increased risk of readmission and revision associated with lumbar decompression and fusion surgery.
